# Birth Preparedness and Its Association with Skilled Birth Attendance and Postpartum Checkups among Mothers in Gibe Wereda, Hadiya Zone, South Ethiopia

**DOI:** 10.1155/2016/6458283

**Published:** 2016-12-27

**Authors:** Yohannes Lakew, Fasil Tessema, Chernet Hailu

**Affiliations:** ^1^Gibe Wereda Health Office, Hadiya Zone, Ethiopia; ^2^Department of Epidemiology, Jimma University, Jimma, Ethiopia

## Abstract

*Background*. Birth preparedness program was designed to enhance skilled birth attendance and postpartum checkups of women in a developing country to reduce the three delays that lead women and neonates to death and disability. However, the relationship between birth preparedness with skilled birth attendance and postpartum checkups among mothers is not well studied. Therefore this study is intended to assess the association between birth preparedness and skilled birth attendance and postpartum checkups.* Methods*. A community based cross-sectional study was conducted from March to April 2014. Eight out of 22 Kebeles were selected using probability proportional to size sampling method. Seven hundred and forty-five mothers were selected randomly from the sampling frame, generated from family folders obtained from health posts. Data was collected using pretested questionnaire by face-to-face interview. Data was entered into EpiData version 3.1 database and analyzed by SPSS version 16.* Result*. Out of 745 sampled mothers 728 (97.7%) participated in the study. One hundred and twelve (15.4%) and 128 (17.6%) mothers got skilled birth attendance and received postpartum checkups for their last child, respectively. Birth preparedness, educational status of women and their husbands, and antenatal care visits of mothers were found to be predictor of skilled birth attendance and postpartum checkups. Mothers well prepared for child birth were 6.7 times more likely to attend skilled birth attendance [AOR = 6.7 (2.7–16.4)] and 3 times more likely to follow postpartum checkups [AOR = 3.0 (1.5–5.9)] than poorly prepared mothers, respectively. Travel time to reach the nearest health facility was found as predictor for postpartum checkups of mothers; mothers who travel ≤ 2 hours were three times more likely to follow postpartum checkups than mothers who travel > 2 hours (AOR (95% CI) = 3.4 (1.5–7.9)).* Conclusion and Recommendation*. Skilled birth attendance and postpartum checkups were low. Encouraging women to attend recommended antenatal care visits and providing advice and education on birth preparedness and obstetric danger signs are important interventions to increase skilled birth attendance and postpartum checkups.

## 1. Background

Child birth is an event celebrated for many thousands of women each day, yet child bearing is an event of maternal and neonatal death and injury occurrence as a result of sudden and unpredictable acute obstetric problems [1–5]. Globally 99% of maternal deaths occur in developing countries in which Ethiopia and nine countries share 60% of deaths [6]. In Ethiopia in 2011 maternal mortality ratio was estimated about 676 deaths per 100,000 life birth which represent 30% of all deaths to women age 15–49 years [7]. Mostly this tragedy occurs during the time of delivery and 48 hours after delivery due to direct obstetric complications [1, 4, 5, 8–11].

Thaddeus and Maine outlined three delays that lead to maternal death and disability; the first delay lies in deciding to seek care when complication occurs; the second is delay in reaching care and the third delay is in receiving care [12]. Nonetheless, the first and second delays can be prevented by timely using skilled birth attendants before labor begins [1, 10, 12–14].

The International Conference on Population and Development (ICPD) (1994), the Fourth World Conference on Women in Beijing (1995), and the Millennium Development Goals (MDG) (2000) recommend skilled birth attendant and postpartum checkups as key intervention to reduce maternal mortality [13–15]. WHO affirms that more than 80% of maternal deaths and disabilities are preventable by skilled birth attendance and following postpartum checkups [1, 4, 5, 16]. However, globally skilled birth attendance was on average 65%; developed countries had over 99% whereas developing countries had 63%, but South Asia and sub-Saharan Africa had less than half and in East Africa 33.7% of women delivered by skilled birth attendant in 2008 [17]. In Ethiopia on 2011 EDHS report, utilization of skilled provider assisted delivery and postnatal care were 10% and 5%, respectively, and Southern Nation, Nationality and People Region skilled attendants assisted delivery and postnatal care were 6.5% and 5%, respectively, which is lower than the coverage of East Africa and developing countries [7]. Also, many studies identified different factors that hinder or laggard the timely utilization of skilled birth attendance and postpartum checkups [18–26], but still the coverage is low. Among those birth preparedness factors was the one that influences skilled birth attendance and postnatal checkups [18–22].

Maternal and Neonatal Health (MNH) Program of JHPIEGO an affiliate of John Hopkins University developed birth preparedness program to tackle the three delays that lead to maternal death. Birth preparedness is the process of planning for normal birth and anticipating the actions needed in case of an emergency. It is a strategy that anticipates, promotes, and motivates women for timely use of SBA and PPC. Timely use of SBA reduces delays in obtaining care and possible occurrence of birth complication and if complication occurs it will be immediately managed. A birth preparedness plan includes identification of the following elements: identifying a skilled birth attendant; identifying the location of the closest appropriate care facility; funds for birth related and emergency expenses; transport to a health facility for the birth and obstetric emergency; arranging clean material for safe delivery and foods; and identification of compatible blood donors in case of emergency complication [8, 10, 11, 27–34].

To the best of our knowledge there was no study conducted that assesses the association between birth preparedness and skilled birth attendant and postpartum period in Ethiopia. This study aimed to assess birth preparedness and its association with skilled birth attendance and postpartum checkups among mothers in south Ethiopia. We hypothesize that well-birth-prepared mothers are more likely to attend skilled birth attendant and follow postpartum care than those poorly prepared.

## 2. Methods and Materials

### 2.1. Study Setting and Sample Size

A community based cross-sectional study was conducted in Gibe Wereda, South Ethiopia. Eight rural Kebeles were selected from 22 using probability proportional to size sampling method. Sampling frame was developed from family folder found at health post from each selected Kebele and all women who gave birth in the last 6 months prior to the study were registered; then sample was allocated proportionally to their size of source population. Finally 745 mothers were selected randomly and interviewed from March to April 2014.

Sample size was calculated using two population proportion formulae using Epi Info version 7 Software based on the following assumptions: expected proportion of skilled birth attendance among birth prepared and non-birth-prepared women were 76.3% and 63.7%, respectively (20), confidence interval 95%, power = 80%, and ratio of 4 : 1 non-birth-prepared to birth prepared mothers. This study included mothers who are permanent residents of the selected Kebeles and mothers who gave birth 6 months preceding the study.

### 2.2. Measurements

A structured questionnaire was adapted from the survey tools. The questionnaire was developed in English version; then it was translated into Amharic and Hadiyissa versions and back-translated to check its consistency in translations by different individuals. Questionnaire was pretested on 38 mothers and findings and experiences from the pretest were utilized to modify the tools. Sociodemographic and reproductive characteristics of mothers were collected using face-to-face interviews by five health extension workers (HEW) in the study participants' homes and two diploma graduate nurses supervising day-to-day activities.

*Skilled Birth Attendance*. WHO defines a skilled birth attendance as delivery attended by an accredited health professional—such as a midwife, doctor, or nurse—who has been educated and trained to proficiency in the skills needed to manage normal (uncomplicated) pregnancies, child birth, and the immediate postnatal period and in the identification, management, and referral of complications in women and newborns; any birth attended by traditional birth attendant, relative or friend, or any other person who is not a health professional with midwifery skills based on the respondent's description was classified as unskilled birth. 

*Postpartum Checkups*. At least a single postpartum checkup during postpartum period, periods beginning after the delivery of the placenta and continuing until 42 days after child birth, is considered as women attended postpartum checkup. 

*Birth Preparedness Index*. Principle Component Analysis (PCA) was used to construct birth preparedness index by using various components suggested by MNH Program of JHPIEGO (identifying skilled attendant, identifying health institution for delivery and complication, saving money, arranging transportation, identifying blood donor, arranged essentials (clean clothes, delivery kit), arranged food, and other arrangements). Birth preparedness level was measured based on eight variables, each variable was assigned a weight (factor score) generated through principal component analysis, and the resulting birth preparedness scores was standardized in relation to a normal distribution with mean zero and standard deviation one. The score distribution was used to categorize birth preparation into poor, moderate, and well. The new variable (birth preparedness index) scale is varying from “poor” to “well” preparedness and further analysis was carried out by this variable. 

*Knowledge of Obstetric Danger Sign*. Women were considered as well knowledgeable about obstetric danger signs related to pregnancy and child birth if they scored equal and above the mean of 17 knowledge questions and if less than mean they were considered as poorly knowledgeable. 

*Wealth Index*. It is a composite measure of the cumulative living standard of a household. The wealth index is calculated using easy-to-collect data on a household's ownership of selected assets, such as television, radio, livestock, furniture, and farm land. Generated with a statistical procedure known as principal components analysis, the wealth index places individual households on a continuous scale of relative wealth. Each household asset for which information is collected is assigned a weight or factor score generated through principal components analysis. The resulting asset scores are standardized in relation to a standard normal distribution with mean zero and standard deviation one. These standardized scores are then used to create the break points that define wealth index as lowest, second, middle, fourth, and highest.

### 2.3. Data Analysis Procedure

Data was cleaned and entered into EpiData version 3.1 database and analyzed using SPSS version 16.0 statistical Software. Bivariate and multivariable logistic regression analysis was used and strength of statistical association was assessed by adjusted odds ratios and 95% confidence intervals and statistical significance were considered at *P* < 0.05.

### 2.4. Ethical Consideration

The study was financially supported and ethical clearance was obtained from Jimma University College of Public Health and Medical Science and permission was also obtained from the local administrative units. Informed verbal consent was obtained from all the study participants.

## 3. Result

A total of 728 participated in the study with response rate of (97.7%). The majority of respondents were between 25 and 34 years of age, married, followers of protestant religion, housewives, and Hadiya by ethnicity and housewives. More than half (53.6%) of the mothers did not attend formal education; 248 (34.1%) attended primary and 90 (12.4%) attended secondary and above education. Almost all respondents (723 (99.3%)) travel on foot and 589 (80.9%) travel greater than two hours to reach the nearest health center. Regarding respondents' husbands' educational and occupational status, 299 (42.1%) did not attend formal education and 542 (76.4%) were farmers. Equal proportion (19.6 to 20.2%) of mothers were in lowest, second, middle, fourth, and highest wealth groups (Table 1).

Regarding previous obstetric characteristics of the mothers, 115 (15.9%) had one and more stillbirth experience and 322 (52.2%) of mothers had experienced at least single obstetric complication. Regarding birth space 368 (59.7%) of mothers were two and less than two years of birth interval between previous and last child and 259 (35.6%) had 2-3 times pregnancies. With respect to previous mode of delivery, 600 (97.1%) had normal delivery (Table 2).

Concerning the last pregnancy characteristics, more than half 423 (58.2%) of mothers planned their last child pregnancy. The majority (563 (77.3%)) attended antenatal visits, but 164 (22.5%) did not attend; 501 (68.8%) of mothers were advised about birth preparedness tools by health professional during their ANC visits and about 118 (16.2%) mothers experienced pregnancy complication. The majority, 613 (84.2%), of mothers delivered at home and 89 (12.2%), 23 (3.2%), and 3 (0.4%) of mothers delivered at health center, hospital, and health post, respectively, and 311 (42.7%) decided their place of delivery by themselves. Out of 728 mothers, 112 (15.4%) of deliveries were assisted by skilled birth attendant (midwifery, nurses, health officer, and medical doctor), and 128 (17.6%) of mothers attended at least one postpartum checkup after their last delivery (Table 3).

As to knowledge on obstetric danger signs, more than half 415 (57.0%) and 313 (43.0%) of the mothers had good knowledge and poor knowledge about danger signs that occur during pregnancy, delivery, and postpartum period, respectively (Table 4).

Pertaining to birth preparedness status of mothers, Table 5 shows the percentage distribution of women in the categories of birth preparation for each tool of birth preparation for their last child birth. Out of 728 mothers 192 (26.4%) identified skilled birth provider, 391 (53.7%) saved money for emergency cost incurred, 20 (2.7%) arranged blood donors, if in case of emergency blood loss during delivery, 272 (37.4%) identified their place of delivery as health institution, 118 (16.2%) arranged their means of transportation during delivery, and 615 (84.5%) arranged delivery kits (clean clothes, cord tie, and new blade); the large majority (684 (94.0%)) prepared foods they consumed after delivery and 105 (14.4%) have made additional arrangement. Using principal component analysis birth preparedness index was constructed to see the level of birth preparedness of the mothers and about 245 (33.6%), 256 (35.2%), and 227 (31.2%) mothers were well, moderately, and poorly prepared, respectively.

As shown in Table 6, multivariable logistic regression analysis was conducted to assess the association between birth preparedness and skilled birth attendance after controlling potential confounding variables. Those well-prepared mothers for child birth were 6.7 times more likely to be assisted by skilled birth attendant than those who prepared poorly [AOR = 6.7 (2.7–16.4)]. Other factors like maternal education were found to be major predictor of skilled birth attendance; mothers whose educational status was primary and secondary and above were 4.7 and 6.2 times more likely to get skilled birth attendant than mothers who did not have formal education [AOR = 4.7 (2.3–9.3) and AOR = 6.3 (2.7–14.7)], respectively. Antenatal care visit had significant association with skilled birth attendance; mothers who attended antenatal care four and above times were 4.8 times more likely to get skilled birth attendant than mothers who did not attend antenatal care [AOR = 4.8 (1.6–15.0)]. Husband's educational status was also significantly associated with skilled birth attendance. Mother's whose husbands' educational status secondary and above were four times more likely to get skilled birth attendant than whose their husbands' educational status was informal education [AOR = 4.0 (1.7–9.3)]. Mothers who experienced two or more stillbirth were six times more likely to get skilled birth attendant than mothers with no history of stillbirth [AOR = 8.8 (2.0–28.7)]. Mothers' who made decision about place of delivery with their husbands were five times more likely to attend skilled birth attendant than those mothers who decided only by themselves [AOR = 4.1 (2.0–8.3)].

As shown in Table 7, birth preparedness was found to be an important predictor of postpartum checkups. Mothers who were well prepared for child birth were 3 times more likely to follow postpartum checkups than who prepared poorly [AOR = 3.0 (1.5–5.9)] after controlling possible confounding variables. There are other variables also predicting postpartum checkups mothers' who had primary and secondary and above educational level being 3.7 and 11.8 times more likely have postpartum checkup than mothers who did not attend formal education [AOR = 3.7 (2.1–6.7) and AOR = 11.8 (2.1–25.3)], respectively.

Husband's educational status was also significantly associated with postpartum checkups of mothers. Mothers whose husbands' educational status secondary and above were 2.1 times more likely to follow postpartum checkups than whose their husbands' educational status was informal education [AOR = 2.1 (1.1–3.3)].

Mothers who attended four or more antenatal care visits and had knowledge of danger signs during pregnancy and child birth were more likely to follow postpartum checkups than mothers who did not attend antenatal care and had poor knowledge [AOR = 3.9 (1.5–10.2)] and [AOR = 1.9 (1.1–3.3)], respectively. Those mothers who travelled ≤ 2 hours from their home to health center were 3.4 times more likely to follow postpartum checkups than those who travelled more than two hours [AOR = 3.4 (1.5–7.9)].

## 4. Discussion

This study assessed the association of birth preparedness with skilled birth attendance and postpartum checkups of mothers in rural area. Birth preparedness becomes predictor of skilled birth attendance and postpartum checkups of mothers. Well-prepared mothers for child birth were more likely to attend skilled delivery and follow postpartum checkups than those poorly prepared. This implies that the birth preparedness components, saving money, arranging transport, identifying skilled birth attendant, and arranging blood donors, are important factors to get health facility. Saving money is important for costs incurred. Arranging transport is important in place where people living rural and far from health facility. Identifying skilled attendant is also important for the psychological confidence and arranging blood donors is also important during emergency bleeding. Therefore, this readiness and preparation for child birth will make mother utilize health service. The plausible reason might be the fact that also when mothers do not prepare well, they bother as labor begins in identifying skilled provider, identifying place of delivery, searching for money for incurred cost, finding transportation, and other things which may contribute to high home delivery. This finding was consistent with the study conducted in Nepal, rural Uganda, and in Madhya Pradesh, India, that revealed prepared mother's use of health facility to get child birth [19, 20, 31, 32].

Educational status of the mothers was significantly associated with skilled birth attendance and postpartum checkups of the mothers. This study was consistent with research done in Ethiopia and Nepal [7, 19, 21, 22, 25]. The possible explanation could be the fact that as mother's educational levels increase the possibility of thinking and analyzing the possible consequence of child birth and also ability to learn and understand health information and education would be high. The level of education is also related to income of the mothers; this will foster the mothers to utilize health services [19, 24].

Another predictor of skilled birth attendance and postpartum checkup was antenatal care of mothers. This could be due to the fact that as mothers attended ANC visits repeatedly they had enough contact time with health professionals which creates good opportunity for advice on birth preparation and on importance of birth preparation, SBA, and PPC. This will empower mothers to seek health service. This study revealed that ANC visit was about 77.7%, but SBA and PPC were very low; it can be considered as missed opportunity; this may indicate that the information, advice, and service delivered to mothers at health institution during ANC visit may be in doubt. This finding was in line with study conducted in parts of Ethiopia [18, 21, 25, 26].

When mothers made decision of place of delivery in consultation with their husbands, the probability of getting birth assisted by a skilled birth attendant was high. However, when women made the final decision alone, the likelihood of getting birth assisted by SBA was low. This might be due to the fact that when mothers decide their place delivery with consultation of their husband, they may get financial and psychological support. Ensuring women's autonomy in deciding place of delivery alone may not guarantee maternal health service utilization. Similarly this finding was consistent with the study done in Southwestern Uganda in 2011 [20]. In contrary this finding was inconsistent with the study conducted in Arsi Zone and Dodota Wereda, Oromia region, in 2011 where mothers who decided their place of delivery by only themselves has higher probability to attend skilled birth attendant [22, 26].

Husbands' educational status was significantly associated with skilled birth attendance and postpartum checkups. Women whose husbands had secondary and above education were more likely to attend skilled birth attendant and postpartum checkups than those who did not attend formal education. This may be possibly a result of the fact that educated husbands might be aware of the danger events occurring during child birth and importance of skilled birth attendance and attending postpartum checkups. This finding was parallel with that of a study done in Munisa Wereda, Oromia region [18].

Knowledge of danger signs during pregnancy and child birth of the mothers was found to be significantly associated with postpartum checkups. Mothers who had good knowledge were more likely to follow postpartum checkups than mothers who had poor knowledge even after controlling the possible confounder. This may be due to the fact that, when mothers were aware of danger signs during pregnancy, they know the possible adverse event happening during postpartum period; this encouraged mothers to follow PPC. This finding was consistent with the study conducted Arsi Zone, Oromia region, in 2011 and Bahirdar, Amhara region, in 2012 [25, 26].

Travel time to reach the nearest health institution was found statistically significant. Mothers who travel ≤ 2 hours were more likely to follow postpartum checkups than mothers travelling > 2 hours to reach the nearest health center. The possible explanation for this is that postnatal period is a period where women's body is sensitive and weak; as a result, physical proximity of health institution was determining factor in case of this study where most of study participants (99%) were travelling on foot. Mothers living in closer proximity to modern health services were more likely to receive services from health personnel for the treatment of life-threatening and high-risk conditions during the postnatal period. This finding was in line with EDHS, 2011, report [7].

Mothers with history of stillbirth were more likely to attend skilled birth attendant than mother who did not experience stillbirth in the study area, even it was statistically significant. This might be due to the fact that mothers who had experienced stillbirth may not want suffering and pain of life loss during child birth; for this reason the possibility of mothers to get skilled birth attendant would be increased among women who had experienced stillbirth than those who had not experienced stillbirth. This result was consistent with the study conducted in in Dodota Wereda, Oromia region [22].

### 4.1. Limitations of This Study

The potential limitations of this study should be considered. The community based sampling strategy, high response rate, and large sample size reduced the possibility of selection bias. Since this study was cross-sectional study there could be temporality bias. However, in preparation of the data collection tool and during interviews, mothers were asked to tell history of exposure status before the occurrence of the outcome. Second, since this study inquires past exposure status of mothers, there could be recall bias. To minimize inaccurate reporting, study participants were selected within six months after delivery. Third, validation of self-report information was not done by comparison to medical records due to inaccessibility of individual files from health facilities. Finally, the influence of specific birth preparedness components on the outcomes was uncertain since, birth preparedness was composite variable derived by PCA.

## 5. Conclusion

In this study percentage of skilled birth attendance and postpartum checkups was very low. However, more than three-fourth of mothers attended antenatal cares. This may create good opportunity by health professionals and health institutions to advise mothers to prepare for child birth and danger signs, attend SBA, and follow PPC. Therefore it is recommended to use this golden opportunity to advocate the importance of skilled delivery and postpartum checkups, and further study should be done on maternal health service providers and health institution characteristics.

## Figures and Tables

**Table 1:** Sociodemographic characteristics of mothers in Gibe Wereda, South Ethiopia, March 2014.

Variables	Category	Number	Percent
Age group of mothers	15–24	194	26.6
	25–34	451	62.0
	≥35	83	11.4
	Mean age 27.91 ± 5.23		

Marital status of mothers	Married	702	96.4
	Single	8	1.1
	Widowed	13	1.8
	Divorced	5	0.7

Religion of mothers	Orthodox	51	7.0
	Catholic	7	1.0
	Protestant	670	92.0

Ethnicity of mothers	Hadiya	665	91.3
	Gurage	38	5.2
	Kembata	14	1.9
	Amhara	11	1.5

Occupation of mothers	Housewife	625	85.9
	Government employee	18	2.5
	Farmer	51	7.0
	Others^*∗∗*^	34	4.7

Educational status of mothers	Informal	390	53.6
	Primary	248	34.1
	Secondary & above	90	12.4

Wealth index	Lowest (Q1)	145	19.9
	Second (Q2)	146	20.1
	Middle (Q3)	145	19.9
	Fourth (Q4)	147	20.2
	Highest (Q5)	145	19.9

Occupation of husband (*N* = 702^*∗*^)	Farmer	537	76.5
	Daily laborer	64	9.1
	Government employee	15	2.1
	Merchant	86	12.3

Educational status of husband (*N* = 702^*∗*^)	Informal	296	42.2
	Primary	318	45.3
	Secondary & above	88	12.9

Family size	≤5	343	47.1
	>5	385	52.9

Time to reach health institute	>2 hours	589	80.9
	≤2 hours	139	19.1

^*∗*^Total not equal to 728 because of missing values.

^*∗∗*^Others include merchant and daily laborer.

**Table 2:** Previous obstetric histories of mothers in Gibe Wereda, South Ethiopia, March 2014.

Variables		Number	Percent (%)
Gravidity	1	111	15.2
	2-3	259	35.6
	4-5	185	25.4
	>5	173	23.8

Stillbirth (*N* = 725^*∗*^)	No stillbirth	610	84.1
	One time only	95	13.1
	≥2	20	2.8

Mode of delivery (*N* = 617^*∗*^)	Cesarean section	17	2.8
	Vaginal	600	97.2

Previous obstetric complication (*N* = 617^*∗*^)	no	295	47.8
	Yes	322	52.2

Birth space (*N* = 617^*∗*^)	≤2	368	59.7
	>2	249	40.3

^*∗*^Total not equal to 728 because of missing values.

**Table 3:** Last pregnancy characteristics of mothers in Gibe Wereda, South Ethiopia, March 2014.

Variables		Number	Percent (%)
Intended pregnancy (*N* = 727^*∗*^)	Yes	423	58.2
	No	304	41.8

ANC visit	None	164	22.5
	One time	93	12.8
	Two times	159	21.8
	Three times	160	22.0
	≥4 times	152	20.9

Last pregnancy complication	Yes	118	16.2
	No	610	83.8

BP advise	No	227	31.2
	Yes	501	68.8

Decision making	Mine only	311	42.7
	My husband only	123	16.9
	Mine & my husband	179	24.6
	Others^*∗∗*^	115	15.8

Place of delivery	Home	613	84.2
	Health post	3	0.4
	Health center	89	12.2
	Hospital	23	3.2

Skilled delivery	Yes	112	15.4
	No	616	84.6

Postpartum checkups	Yes	128	17.6
	No	600	82.4

^*∗*^Total not equal to 728 because of missing values.

^*∗∗*^Others include family and neighbors.

**Table 4:** Knowledge of danger signs during pregnancy and child birth of mothers in Gibe Wereda, South Ethiopia, March 2014.

Variables	Not knowledgeable *N* (%)	Knowledgeable *N* (%)
Knowledge of danger signs during pregnancy		
Persistent vomiting	186 (25.5)	542 (74.5)
Vaginal bleeding	123 (16.9)	605 (83.1)
Severe headache	533 (73.2)	195 (26.8)
Hypertension	543 (74.6)	185 (25.4)
Face, hand, and feet swelling	322 (44.2)	406 (55.8)
Knowledge of danger signs during delivery		
Prolonged labor (>12 hours)	116 (15.9)	612 (84.1)
Retained placenta	122 (16.8)	606 (83.2)
Vaginal bleeding	139 (19.1)	589 (80.9)
Hypertension	576 (79.1)	15 (20.9)
Knowledge of danger signs during postpartum period		
Massive vaginal bleeding	84 (11.5)	644 (88.5)
After pain	131 (18.0)	597 (82.0)
High-grade fever	502 (69.0)	226 (31.0)
Offensive vaginal discharge	344 (47.3)	384 (52.7)
Hypertension	510 (70.1)	218 (29.9)
Face, hand, and feet swelling	352 (48.4)	376 (51.6)

Overall knowledge on danger signs during pregnancy and child birth	313 (43.0)^*∗*^	415 (57.0)^*∗∗*^

^*∗*^Poor knowledge about danger signs.

^*∗∗*^Good knowledge about danger signs.

**Table 5:** Percentage distributions of mothers in the categories of birth preparation tools of mothers in Gibe Wereda, South Ethiopia, March 2014.

Birth preparedness	Response categories			
	Yes		No	
	*N*	%	*N*	%
Identified skilled provider	192	26.4	536	73.6
Saved money	391	53.7	337	46.3
Arrange blood donor	20	2.7	708	97.3
Identified place of delivery	272	37.4	456	62.6
Arrange transport	118	16.2	610	83.8
Prepared delivery kit	615	84.5	113	15.5
Prepared food	684	94	44	6
Made other arrangements	105	14.4	623	85.6

**Table 6:** Logistic regression of birth preparedness and its association with skilled birth attendance in Gibe Wereda, South Ethiopia, March 2014.

Variables	Skilled birth attendance		Odds ratio	
	No (%)	Yes (%)	COR (95%)	AOR (95%)
Birth preparedness index				
Poor	217 (29.8)	10 (1.4)	1.0	1.0
Moderate	240 (33.0)	16 (2.2)	1.4 (0.6–3.2)	1.3 (0.5–3.5)
Well	159 (21.8)	86 (11.8)	11.7 (5.9–23.3)	6.7 (2.7–16.4)^*∗*^
Mother educational status				
Informal	366 (50.3)	24 (3.3)	1.0	1.0
Primary	202 (27.7)	46 (6.3)	3.5 (2.1–5.8)	4.7 (2.3–9.3)^*∗*^
Secondary & above	48 (6.6)	42 (5.8)	13.3 (7.4–23.9)	6.3 (2.7–14.7)^*∗*^
Mother occupational status				
Housewife	539 (74.0)	86 (11.8)	1.0	1.0
Government employee	5 (0.7)	13 (1.8)	16.3 (5.6–46.8)	9.8 (0.9–29.9)
Farmer	45 (6.2)	6 (0.8)	0.8 (0.3–2.0)	1.0 (0.3–3.8)
Other^*∗∗*^	27 (3.7)	7 (1.0)	1.6 (0.7–3.8)	0.9 (0.3–2.8)
Travel time				
>2 hr	539 (74.0)	86 (11.8)	2.7 (1.4–5.3)	1.8 (0.7–4.3)
≤2 hr	5 (0.7)	13 (1.8)	1.0	1.0
Husband educational status (*N* = 702)				
No formal	277 (39.4)	19 (2.7)	1.0	1.0
Primary	270 (38.4)	48 (6.8)	2.6 (1.5–4.5)	1.8 (0.9–3.7)
Secondary & above	44 (6.3)	44 (6.3)	14.5 (7.8–27.2)	4.0 (1.7–9.3)^*∗*^
Gravidity				
1	84 (11.5)	27 (3.7)	2.3 (1.2–4.4)	0.4 (0.1–1.2)
2-3	217 (29.8)	42 (5.8)	1.4 (0.8–2.5)	0.4 (0.2–1.0)
4-5	163 (22.4)	22 (3.0)	1.0 (0.5–1.8)	0.6 (0.2–1.4)
>5	152 (20.9)	21 (2.9)	1.0	1.0
Stillbirth (*N* = 725)				
No	518 (71.4)	92 (12.7)	1.0	1.00
One	84 (11.6)	11 (1.5)	0.7 (0.4–1.4)	1.3 (0.5–3.4)
2 and above	13 (1.8)	7 (1.0)	3.0 (1.2–7.8)	8.8 (2.0–28.7)^*∗*^
Intend last pregnancy (*N* = 727)				
No	272 (37.4)	32 (4.4)	1.0	1.00
Yes	343 (47.2)	80 (11.0)	2.0 (1.3–3.1)	0.7 (0.4–1.4)
ANC visit				
No ANC visit	158 (21.7)	6 (0.8)	1.0	1.0
One time	86 (11.8)	7 (1.0)	2.1 (0.7–6.6)	1.4 (0.4–5.3)
Two times	151 (20.7)	8 (1.1)	1.4 (0.5–4.1)	1.2 (0.4–4.2)
Three times	132 (18.1)	28 (3.8)	5.6 (2.2–13.9)	2.4 (0.8–7.4)
4 & above	89 (12.2)	63 (8.7)	18.6 (7.7–44.8)	4.8 (1.6–15.0)^*∗*^
Knowledge of danger signs during pregnancy & child birth				
Poor knowledgeability	280 (38.5)	33 (4.5)	1.0	1.0
Good Knowledgeability	336 (46.2)	79 (10.9)	2.0 (1.3–3.1)	1.7 (0.9–3.1)
Last pregnancy complication				
No	524 (72.0)	86 (11.8)	1.0	1.0
Yes	92 (12.6)	26 (3.6)	1.7 (1.1–2.8)	1.2 (0.6–2.5)
Decision making				
Herself only	289 (39.7)	22 (3.0)	1.0	1.0
Husband only	109 (15.0)	14 (1.9)	1.7 (0.8–3.4)	1.8 (0.7–4.4)
Herself & husband	119 (16.3)	60 (8.2)	6.6 (3.9–11.3)	4.1 (2.0–8.3)^*∗*^
Family/relatives	99 (13.6)	16 (2.2)	2.1 (1.1–4.2)	1.4 (0.6–3.6)

^*∗*^Significant at *P* value < 0.05.

^*∗∗*^Other—merchant and daily laborer.

**Table 7:** Logistic regression of birth preparedness and its association with postpartum checkups in Gibe Wereda, South Ethiopia, March 2014.

Variables	Postpartum checkups		Odds ratio	
	No	Yes	COR (95%)	AOR (95%)
Birth preparedness index				
Poor	209 (28.7)	18 (2.5)	1.0	1.0
Moderate	232 (31.9)	24 (3.3)	1.2 (0.6–2.3)	0.8 (0.4–1.7)
Well	159 (21.8)	86 (11.8)	6.3 (3.6–10.8)	3.0 (1.5–5.9)^*∗*^
Mother education				
Informal	362 (49.7)	28 (3.8)	1.0	1.0
Primary	198 (27.2)	50 (6.9)	3.3 (2.0–5.4)	3.7 (2.1–6.7)^*∗*^
Secondary and above	40 (5.5)	50 (6.9)	16.1 (9.2–28.5)	11.8 (5.5–25.3)^*∗*^
Mother occupation				
Housewife	522 (71.7)	103 (14.1)	1.0	1.0
Government employee	6 (0.8)	12 (1.6)	10.1 (3.7–27.6)	3.9 (0.8–20.3)
Farmer	44 (6.0)	7 (1.0)	0.8 (0.3–1.8)	1.4 (0.5–4.1)
Other^*∗∗*^	28 (3.8)	6 (0.8)	1.1 (0.4–2.7)	0.5 (0.2–1.8)
Wealth index				
Lowest	102 (14.0)	43 (5.9)	1.0	1.0
Second	131 (18.0)	15 (2.1)	0.7 (0.4–1.3)	0.4 (0.2–1.2)
Middle	120 (16.5)	25 (3.4)	0.7 (0.4–1.4)	0.9 (0.4–2.0)
Fourth	131 (18.0)	16 (2.2)	0.5 (0.3–1.0)	0.8 (0.3–1.8)
Highest	116 (15.9)	29 (4.0)	0.9 (0.5–1.6)	1.3 (0.6–2.7)
Travel time				
≤2 hr	130 (17.9)	9 (1.2)	3.6 (1.8–7.4)	3.4 (1.5–7.9)^*∗*^
>2 hr	470 (64.6)	119 (16.3)	1.0	1.0
Husband education (*N* = 702)				
Informal	268 (38.2)	28 (3.9)	1.0	1.0
Primary	261 (37.2)	57 (8.2)	2.1 (1.3–3.4)	1.4 (0.8–2.4)
Secondary and above	46 (6.5)	42 (6.0)	8.7 (4.9–15.5)	2.1 (1.1–4.8)^*∗*^
Family size				
>5	334 (45.9)	51 (51.0)	1.9 (1.3–2.8)	1.6 (0.7–3.7)
≤5	266 (36.5)	77 (10.6)	1.0	1.0
Gravidity				
1	82 (11.3)	29 (4.0)	2.7 (1.4–5.0)	0.4 (0.1–1.3)
2-3	206 (28.3)	53 (7.3)	1.9 (1.1–3.4)	0.6 (0.2–1.8)
4-5	159 (21.8)	26 (3.6)	1.3 (0.6–2.3)	0.8 (0.4–1.9)
>5	153 (21.0)	20 (2.7)	1.0	1.0
Still birth (*N* = 725)				
No	503 (69.4)	107 (14.8)	1.0	1.0
One	82 (11.3)	13 (1.8)	0.7 (0.4–1.4)	1.1 (0.5–2.4)
Two and above	14 (1.9)	6 (0.8)	2.0 (0.7–5.4)	2.8 (0.8–10.1)
Intend pregnancy (*N* = 727)				
No	263 (36.2)	41 (5.6)	1.0	1.0
Yes	336 (46.2)	87 (12.0)	1.6 (1.1–2.4)	0.7 (0.4–1.2)
ANC visit				
No ANC	155 (21.3)	9 (1.2)	1.0	1.0
One time	83 (11.4)	10 (1.4)	2.1 (0.8–5.3)	1.5 (0.5–4.6)
Two times	144 (19.8)	15 (2.1)	1.8 (0.7–4.2)	1.6 (0.6–4.4)
Three times	129 (17.7)	31 (4.3)	4.1 (1.9–9.0)	1.9 (0.7–4.8)
4 & above times	89 (12.2)	63 (8.7)	12.2 (5.7–25.7)	3.9 (1.5–10.2)^*∗*^
Knowledge of danger signs during pregnancy & childbirth				
Poor knowledgeability	276 (37.9)	37 (5.1)	1.0	1.0
Good Knowledgeability	324 (44.5)	91 (12.5)	2.1 (1.4–3.1)	1.9 (1.1–3.3)^*∗*^
Last pregnancy complication				
No	515 (70.7)	95 (13.1)	1.0	1.0
Yes	85 (11.7)	33 (4.5)	2.1 (1.3–3.3)	1.8 (0.9–3.4)

^*∗*^Significant at *P* value < 0.05.

^*∗∗*^Other—merchant and daily laborer.
